# Sick leave one year after COVID-19 infection: a nationwide cohort study during the first wave in Sweden

**DOI:** 10.1038/s41598-023-50413-9

**Published:** 2024-01-05

**Authors:** Tamar Abzhandadze, Emma Westerlind, Annie Palstam, Katharina S. Sunnerhagen, Hanna C. Persson

**Affiliations:** 1 Institute of Neuroscience and Physiology, The Sahlgrenska Academy, University of Gothenburg, Gothenburg, Sweden; 2 Department of Occupational Therapy and Physiotherapy, Sahlgrenska University Hospital, Gothenburg, Sweden; 3 Department of Emergency Medicine, Sahlgrenska University Hospital, Gothenburg, Sweden; 4 Department of Neurocare, Sahlgrenska University Hospital, Gothenburg, Sweden; 5 School of Health and Welfare, Dalarna University, Falun, Sweden

**Keywords:** Diseases, Health care

## Abstract

This study aimed to investigate the patterns of sick leave, as well as factors associated with sick leave due to COVID-19 during one year after the COVID-19 diagnosis, and sex-related aspects on sick leave. This nationwide study involved 11,902 individuals who received sickness benefits for COVID-19 during the first wave of the pandemic. Data from three Swedish registries were analyzed for sick leave that commenced between March 1 and August 31, 2020, with a follow-up period of 12 months. Sick leave due to COVID-19 was counted as the number of days with sickness benefits and required to include at least one registered COVID-19 diagnosis. The median duration of sick leave was 35 days, and 347 (2.9%) individuals continued their sick leave during the entire follow-up period. Furthermore, 1 year later, the cumulative incidence of sick leave was slightly higher in males (3.5%) compared to females (2.7%). Older age, being single with no children, diagnosed with the virus, medium income level, history of sick leave, and need for inpatient care were significantly associated with a higher duration of sick leave due to COVID-19, both in the total population and when stratified by sex. These results indicated that three out of 100 (3%) patients were still on sick leave 1 year after their COVID-19 diagnosis. Aspects regarding the importance of sick leave duration differed between males and females and comprised sociodemographic characteristics and need for inpatient care. The results indicated the complexity of sick leave due to COVID-19.

## Introduction

The coronavirus disease 2019 (COVID-19) infection caused by the severe acute respiratory syndrome coronavirus 2 (SARS-CoV-2) virus posed a significant challenge for healthcare and the public system^1^. COVID-19 caused long-term consequences in many areas, including activity and participation limitation, psychological functioning, and manifestations in the central nervous, cardiovascular, and pulmonary systems^1–5^. Collectively, these negatively affect an individual’s health.

Sick leave is an indicator of poor self-reported health, mild psychiatric morbidity, long-term physical illness, disability, and mortality^6^. In Sweden, the employer pays for the first two weeks of sick leave. Thereafter, sickness benefits are paid by the tax-financed Swedish Social Insurance Agency (SSIA). The SSIA is a public authority that administers financial compensation during sickness absence to those with all types of employment, on parental leave, or unemployed. The SSIA uses a model that becomes more stringent as absence from work increases. The SSIA granted 601,667 and 591,028 individuals (approximately 6% of the Swedish population) sickness benefits in 2018 and 2019, respectively^7,8^. However, during the first pandemic year, a sharp increase was observed as 680,000 people were granted sickness benefits in 2020^9^.

We demonstrated that 9% of individuals who had taken sick leave due to COVID-19 (during the first wave in Sweden, spanning from March 1 to August 31), were still on sick leave at the end of a four-month follow-up^10^. In a large cohort of public employees, approximately one-third reported problems regarding work ability due to COVID-19^2^. In addition, sex, old age, severe COVID-19 infection, and a prior history of sick leave were associated with COVID-19 related sick leave early after an infection^10–12^. However, the long-term consequences of COVID-19 on sick leave remain unclear.

Previous studies also reported that females were more likely to report a greater degree of disability during sub-acute and post COVID-19^2,13–15^. Meanwhile, males were more likely to have more severe COVID-19 and require inpatient care^2,13–15^. In Sweden, sex differences were reported regarding the overall number of people who received sickness benefits, with a higher proportion among females^7–9^. Sex differences were related to sick leave duration during 1–24 weeks after COVID-19 infection^16^. Higher duration of sick leave was related to older age and severity of COVID-19 infection in females and males, respectively^16^. However, the additional factors that contribute to prolonged sick leave, as well as patterns of sick leave in a longer follow-up period after COVID-19 infection remain unclear.

This study aimed to investigate the patterns of sick leave, as well as factors associated with sick leave due to COVID-19 during one year after the COVID-19 diagnosis, and sex-related aspects on sick leave.

## Methods

### Study design and individuals

In this nationwide registry study, data from three Swedish registries were analyzed. The SSIA provided data on employment type, employment status, and sick leave 1 year prior to COVID-19, as well as the first year after the COVID-19 pandemic. The National Board of Health and Welfare provided data on date of death during the study period. Data from the National Patient Register, which includes all inpatient care in Sweden, were used to investigate hospital stay due to Covid-19. Statistics Sweden provided data on sociodemographic variables. A unique Swedish personal identification number was used to pool data. Data files were pseudonymized and contained serial numbers used to merge them. The Swedish National Board of Health and Welfare maintained the code key for the serial numbers.

The inclusion criteria were residents of Sweden aged ≥ 18 years who received sickness benefits due to COVID-19 (defined according to the International Classification of Diseases (ICD) codes U 07.1 or 07.2). Sick leaves were required to have commenced between March 1 and August 31, 2020 (which corresponded to the first wave in Sweden), with a follow-up period of 12 months. Data were excluded if individuals had unspecified diagnoses (ICD codes Z, n = 27), died during the study period (n = 30), or had a primary diagnosis based on ICD codes that indicated diseases of uncertain etiology (n = 23).

### Outcomes

The outcome was a count variable of the duration of sick leave due to COVID-19 as registered by the SSIA. When followed-up the sick leave period was required to include at least one sick leave due to a COVID-19 (confirmed by laboratory tests and assigned the ICD code U 07.1. If virus was not identified, the ICD code U 07.2 was assigned). However, it could be combined with sick leave due to other diagnoses if the gap between them was less than two weeks and the other diagnosis was deemed related to COVID-19. Such related diagnoses could include viral infections, fever, or a second sick leave registration due to COVID-19. The list of related diagnoses was provided by Westerlind et al.^10^ in the Supplementary Table 1. If the gap in non-registration between sick leave periods was ≤ 2 weeks, it was regarded as one period. In accordance with the Swedish social insurance regulations, work ability is primarily related to current employment status for up to 180 days of sick leave. From ≥ 181 days, it is related to the general labor market.

### Explanatory variables

Sick leave prior to COVID-19 was defined as either being on sick leave for at least 28 days or at least six times between March 1, 2019, and the start of the study period (March 1, 2020).

Employment status at the start of sick leave due to COVID-19 was categorized as employment (which included on parental leave, combined employment), self-employment, or unemployment (which included those who studied).

In the year prior to COVID-19, educational level was categorized as primary school (≤ 9 years), secondary school (10–12 years), short university education (13–14 years), or long university education (≥ 15 years). In the regression analyses, the variable was dichotomized as had an education of ≤ 12 years or ≥ 13 years.

Income, in the year prior to COVID-19 (2019), was counted as each person’s disposable income in Swedish Krona (1 Euro = 9.94 SEK, October 29, 2021). To simplify the interpretation, the variable was analyzed in tertiles.

Countries of birth were categorized as Sweden, Nordic countries, except Sweden, European countries, except Nordic countries, or countries outside Europe. In the regression analyses, the variable was dichotomized as Sweden or all other countries.

Familial status, in the year of COVID-19 (2020), was presented as married (which comprised of both married and registered partnership) with children, married without children, single with children, or single without children.

Inpatient care due to COVID-19 was classified as more than 1 day of hospital stay with registration of COVID-19 (U07) as either the primary or secondary diagnosis^10^.

### Statistics

To investigate the patterns of sick leave, Kaplan–Meier curves were used to graphically present the cumulative incidence of sick leave over time. No censoring was applied.

### Regression analysis

**Figure 1:**
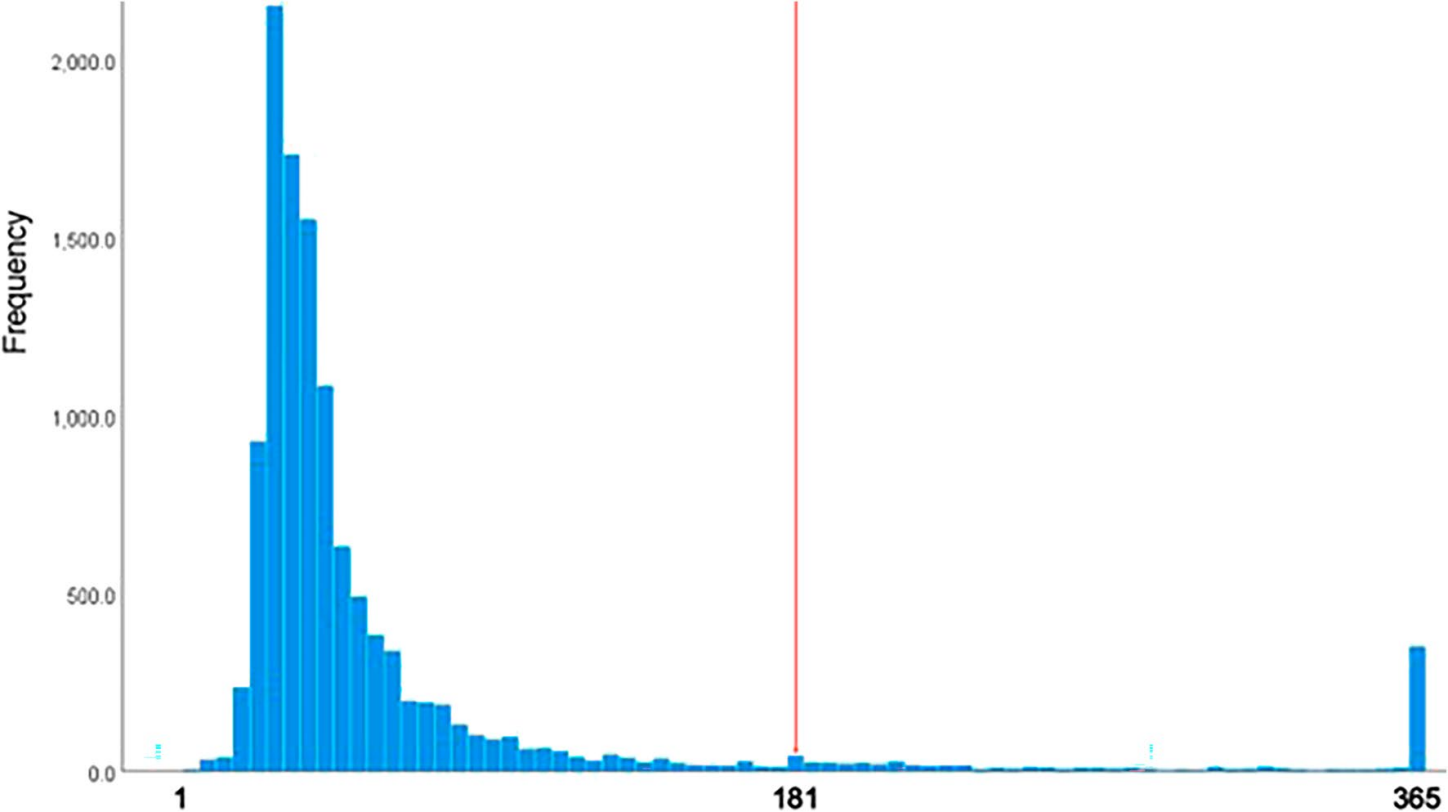
Distribution of sick leave days during one year after the first wave of the COVID-19 pandemic.

A regression analysis was performed to identify factors that explain the duration of sick leave. The outcome was a count variable: sick leave duration during the 1-year period after the first wave of COVID-19. Examination of the variable distribution revealed a small peak at 181 days, long tail ≥ 181 days, and another peak at 365 days at the end of the follow-up (Fig. 1). The peak observed at 181 days could be explained by the Swedish regulations, as described in the outcomes. The second peak at 365 days could be explained by the fact that the follow-up period finished at this time point. The regression model was fitted with the total data from 1 to 356 days.

Subgroup analyses were performed with data on sick leave durations of 1–180 days. We anticipated that having a single data point with such a pronounced peak might introduce noise into the model and potentially influence the model parameters. Simultaneously, our objective was to investigate the factors influencing sick leave duration during the 12 months following the initial COVID-19 diagnosis. Therefore, to mitigate potential noise and ensure a more focused analysis, we conducted a subgroup analysis specifically for the 1–180-day duration range.

The mean and variance were examined to select the proper regression analyses. The mean was less than the variance, indicating that negative binomial regression was a more suitable approach^17^. The explanatory variables were age, employment status, education/dichotomized, country of birth/dichotomized, familial status, COVID-19 diagnosis, and need for inpatient care. These explanatory variables were selected based on previous studies^2,10–12,16,18^. In addition, we conducted interaction analyses on the total sample to examine the potential moderating effects of sex on the relationship between each explanatory variable and the outcome. The identification of statistically significant interactions indicated that sex plays an important role in influencing how these explanatory variables relate to the outcome. Moreover, the models were stratified for both males and females.

Negative binomial regression analysis was performed to predict sick leave duration. To ensure the utmost robustness and reliability of our models, the dataset was randomly divided into two subsets: a training set (80%) and a testing set (20%). We wanted to access the model's proficiency in generalization and its capacity to provide precise predictions for unseen data. For the validation parameters, a simple bootstrapping method was chosen with n = 500 random resamples. The negative binomial regression model was fitted to the training data via the validation parameters. The trained model was evaluated regarding the root mean square error (RMSE) and R-squared values. The models were further fitted to the testing set and the values were evaluated. In addition, to investigate sex-related differences in the factors that explained the duration of sick leave, the effect of modifying sex on independent variables by including interaction terms in the regression models was investigated.

Dispersion parameter (SE) and Akaike information criteria (AIC) were reported for all regression models. Results at the variable level were evaluated using beta coefficients (β), 95% confidence intervals (CI) for OR, and p-values.

Missing data: The number (n) and percentage (%) of missing data per variable were reported. The observed percentage of missingness in regression models ranged from 0.5 to 1.5%. Hence, we made the decision not to impute data due to the low frequency of missing values.

Data were analyzed using SPSS (IBM SPSS Statistics for Windows, Version 28.0. Armonk, NY) and R software (R Core Team, version 4.0.2). The significance level for two-tailed tests was set at an alpha level of 5%.

### Ethics

This study was approved by the Swedish Ethical Review Authority (2020–03046, amendment 2020–03922). Collected data were protected by confidentiality regulations for Public Access to Information. Secrecy Act (2009:400), Chapter 24. The data were handled in accordance with the European Union (EU) General Data Protection Regulation (GDPR) and Swedish law (2018:218), which supplemented the GDPR. The Declaration of Helsinki was not relevant to this project, which was based on data generated in public registries.

### Informed consent

According to the Swedish Ethical Review Authority, quality registers are exempt to the general rule of patient consent according to the Personal Data Act (Swedish law No. SFS 1998:204).

## Results

### Study population

The study population comprised 11,902 individuals (Table 1). The median age was 47.9 years, 40% were males, and 25% required inpatient care due to COVID-19.

**Table 1:** Characteristics of the study population.

	Total sample n = 11,902	Male n = 4792	Female n = 7110
Sociodemographic characteristics			
Age in years, mean (SD)	47.9 (11.3)	48.3 (11.7)	47.8 (11.0)
Median, (IQR [min–max])	50 (17 [18–77])	50 (17 [18–77])	49 (16 [19–76])
Country of birth, n (%)			
Sweden	7517 (63)	2788 (58)	4729 (67)
Nordic countries, except for Sweden	269 (2)	84 (2)	185 (3)
European countries, except for the Nordic countries	1199 (10)	536 (11)	663 (9)
Countries outside of Europe	2908 (25)	1379 (29)	1529 (21)
Educational level, n (%)			
Primary school (≤ 9 years)	1228 (10)	692 (14)	536 (8)
Secondary school (10–12 years)	5866 (50)	2467 (52)	3399 (48)
Short university education (13–14 years)	1736 (15)	718 (15)	1018 (14)
Long university education (≥ 15 years)	2982 (25)	856 (18)	2126 (30)
Income, in 1000 SEK, n (%)			
Low income	3971 (33)	1320 (28)	2651 (37)
Medium income	3967 (33)	1449 (30)	2518 (35)
High income	3962 (33)	2023 (42)	1939 (27)
Familial status, n (%)			
Married, no children	1950 (16)	745 (16)	1205 (17)
Married, with children	4935 (42)	2070 (43)	2865 (40)
Single, with children	1567 (13)	316 (7)	1251 (18)
Single, no children	3442 (29)	1655 (35)	1787 (25)
Sick leave prior to COVID-19, n (%)			
Yes, ≥ 28 days or ≥ 6 times	1927 (16)	565 (12)	1362 (19)
Employment status, n (%)			
Employed	11,408 (96)	4497 (94)	6911(97)
Self-employment	287 (2)	190 (4)	97 (1)
Unemployed	204 (2)	103 (2)	101 (2)
COVID-19 related characteristics			
Diagnosis type			
SARS-CoV-2 detected, U07.1	7955 (67)	3232 (67)	4723 (66)
SARS-CoV-2 not detected, U07.2	3947 (33)	1560 (33)	2387 (34)
Required inpatient care due to COVID-19			
Yes	2931 (25)	1870 (39)	1061 (15)
No	8971 (75)	2922 (61)	6049 (85)
Duration of sick leave due to COVID-19, days			
Mean (SD)	59.4 (70)	64.2 (76.5)	56.2 (65.5)
Median, (IQR [min–max])	35 (26 [1–365])	36 (30 [1–365])	35 (24 [2–365])
Continued sick leave for 12 months, n (%)			
Yes	347 (3)	163 (3)	184 (3)
No	11,555 (97)	4629 (97)	6926 (97)

*Note*: s.d, standard deviation; IQR, interquartile range; Min–max, minimum—maximum; COVID-19, novel SARS-CoV-2 infection. Variables with missing values, n: Country of birth, 9; Educational level, 90; Income, 2; Familial status, 8; Employment status, 3.

### Sick leave patterns during 12 months after COVID-19 onset

**Figure 2:**
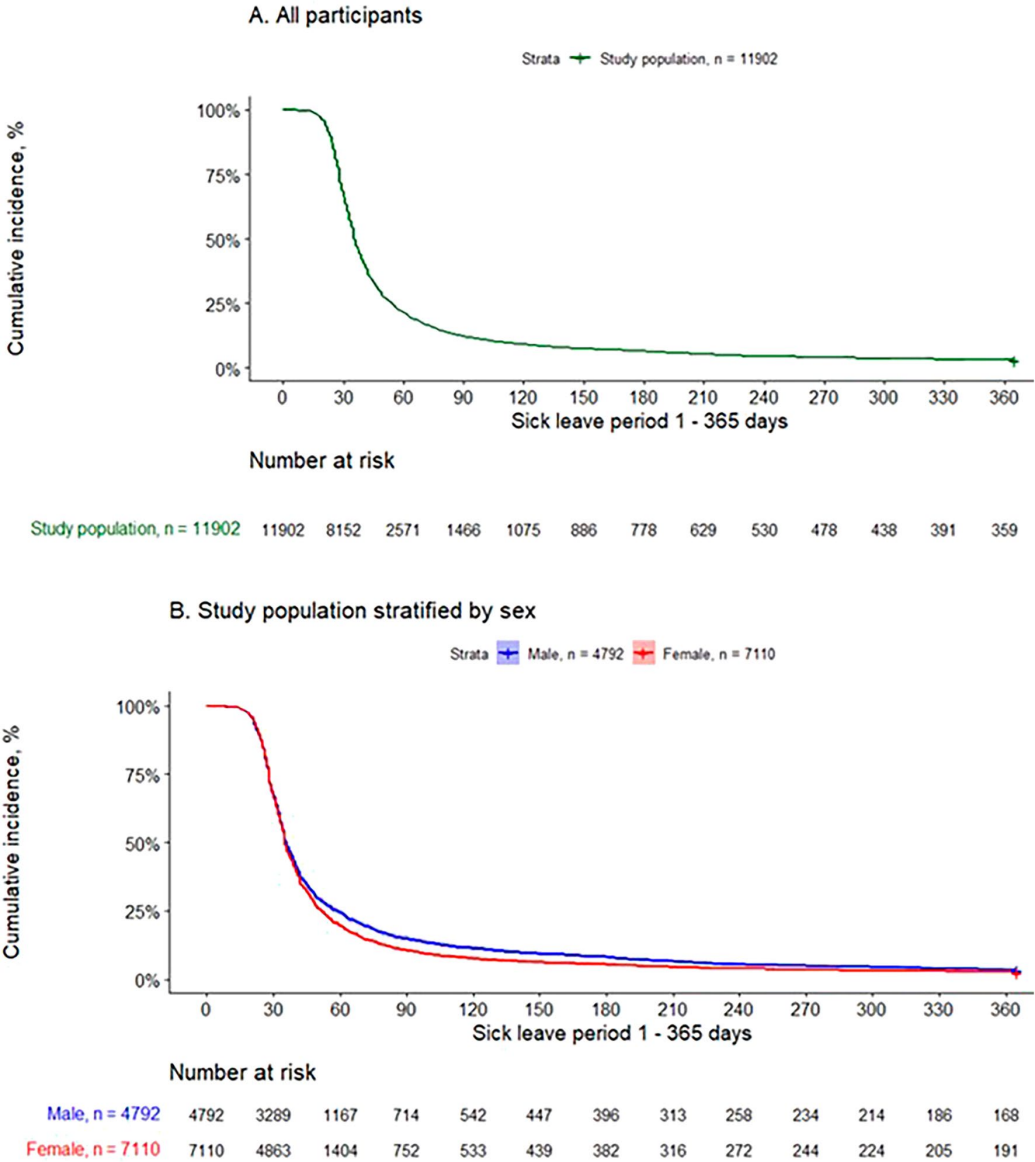
Kaplan–Meier curves showing the cumulative incidence of sick leave during 12 months after COVID-19 diagnosis; (**A**) the total study population; (**B**) stratified by sex.

The median duration of sick leave due to COVID-19 was 35 days (mean [SD], 59.4 [70] days) (Table 1). Moreover, 347 (2.9%) individuals continued their sick leave during the entire follow-up period of 12 months (Fig. 2A). Furthermore, the cumulative incidence was slightly higher in males (3.5%) compared to females (2.7%) (Fig. 2B).

### Prediction of sick leave duration (range 1–365 days)

Odds for longer sick leave increased if individuals were self-employed (OR 1.09), unemployed (OR 1.19), older (OR 1.01 per gained year), single with no children (OR 1.08), single with children (OR 1.06), had been on sick leave before COVID-19 (OR 1.22), had an education of ≥ 13 years (OR 1.06), required inpatient care (OR 1.92), and of the female sex (OR 1.06) (Table 2). Moreover, the odds decreased for individuals born outside Sweden (OR, 0.94) and those without laboratory confirmed COVID-19 virus (ICD code, U 07.2 [OR, 0.85]).

**Table 2:** Multivariable negative binomial regression model explaining the duration of sick leave (range 1–365 days) during first year after COVID-19 infection in the total population (n = 11 799^#^).

Variables	B	SE	OR	95% CI		Adjusted p-value
Ref. Employed						
Self-employment	0.09	0.05	1.09	0.99	1.21	0.052
Unemployed	0.17	0.06	1.19	1.05	1.33	0.003
Age (Range 18–77 years)	0.01	0.00	1.01	1.00	1.01	< 0.001
Ref. Country of birth, Sweden						
All other countries	−0.06	0.01	0.94	0.92	0.96	< 0.001
Ref. Familial status, married no children						
Married, with children	0.02	0.02	1.02	0.98	1.05	0.244
Single, with children	0.06	0.03	1.06	1.00	1.13	0.026
Single, no children	0.08	0.02	1.08	1.00	1.13	< 0.001
Ref. SARS-CoV-2 detected, U07.1						
SARS-CoV-2 not detected, U07.2	− 0.16	0.02	0.85	0.82	0.89	< 0.001
Ref. Low income						
Medium income	− 0.01	0.01	0.99	0.96	1.02	0.357
High income	0.00	0.01	1.00	0.97	1.02	0.985
Ref. No sick leave prior to COVID-19						
Sick leave prior to COVID-19, ≥ 28 days or ≥ 6 times	0.20	0.02	1.22	1.17	1.27	< 0.001
Ref. Education, ≤ 12 years						
Education, ≥ 13 years	0.06	0.02	1.06	1.02	1.10	< 0.001
Ref. No inpatient care						
Inpatient care	0.65	0.02	1.92	1.84	1.99	< 0.001
Ref. Male sex						
Female	0.06	0.02	1.06	1.02	1.10	< 0.001

*Note*: Statistics: negative binomial regression model developed on the test dataset. The dispersion parameter for negative binomial model, theta [SE], 1.98 [0.03]); Akaike information criterion 93,253; RMSE = 67.4; Variance of the model (R-squared) = 0.07. Testing dataset: RMSE = 70.0; R-squared = 0.05. SARS-CoV-2, COVID-19 virus infection; Bootstrapped estimates: B, regression coefficient; SE, Standard Error; OR, Odds ratio; 95% CI, 95% confidence interval for odds ratio.

^#^The population size in regression analysis was 11,799 individuals due to missing data on explanatory variables.

### Sex-related aspects in regarding sick leave duration (range 1–365 days)

Significant interactions were found between sex, age, country of birth, family status, type fo COVID-19 ICD-10 codes, income, education level, and inpatient care (Table 3). Country of birth outside Sweden, being single with or without children, having a high-income level, and an education of ≥ 13 years were significantly associated with longer sick leave duration in females; however, not in males (Table 3).

**Table 3:** Individual negative binomial regression analyses for males and females.

Explanatory variables	Males (n = 4723)^#^		Females (n = 7076)^#^		Total sample (n = 11 799)^#^	
	OR (95% CI)		OR (95% CI)		95% CI *Interaction*	p-Value *Interaction*
Ref. Employed						
Self-employment	1.01	(0.88–1.16)	1.17	(0.95–1.46)	(0.87–1.29)	0.569
Unemployed	1.07	(0.86–1.33)	1.19	(0.96–1.47)	(0.86–1.32)	0.622
Age (Range 18–77 years)	1.01	(1.00–1.01)***	1.00	(1.00–1.00)*	(1.00–1.00)	< 0.001
Ref. Country of birth, Sweden						
All other countries	0.99	(0.93–1.05)	0.91	(0.86–0.97)**	(0.85–0.96)	0.002
Ref. Familial status, married no children						
Married, with children	0.96	(0.87–1.06)	1.06	(0.98–1.15)	(1.10–1.28)	< 0.001
Single, with children	0.98	(0.85–1.12)	1.13	(1.02–1.24)*	(1.16–1.47)	< 0.001
Single, no children	1.01	(0.92–1.11)	1.13	(1.04–1.22)**	(1.11–1.35)	< 0.001
Ref. SARS-CoV-2 detected, U07.1						
SARS-CoV-2 not detected, U07.2	0.91	(0.85–0.99)*	0.84	(0.80–0.89)***	(0.83–0.93)	< 0.001
Ref. Low income						
Medium income	0.95	(0.90–1.01)	1.02	(0.96–1.08)	(1.00–1.13)	0.027
High income	0.99	(0.93–1.05)	1.05	(1.01–1.09)*	(1.04–1.17)	< 0.001
Ref. No sick leave before COVID-19						
Sick leave before COVID-19, ≥ 28 d or ≥ 6 t	1.26	(1.16–1.36)***	1.19	(1.12–1.26)***	(0.92–1.07)	0.798
Ref. Education, ≤ 12 years						
Education, ≥ 13 years	0.99	(0.93–1.05)	1.12	(1.05–1.18)***	(1.07–1.21)	< 0.001
Ref. No inpatient care						
Inpatient care	1.95	(1.81–2.11)***	1.72	(1.59–1.86)***	(0.78–0.91)	< 0.001

Interaction analyses on effects of sex for explaining sick leave duration (range 1–365 days) during 1 year after the first wave.

*Note*: SARS-CoV-2, COVID-19 virus infection; OR, Odds ratio; 95% CI, 95% confidence interval for odds ratio. ***p < 0.001, **p < 0.01, *p < 0.05. Statistics: negative binomial regression model. ^#^The number of individuals is different due to missing data on explanatory variables.

Model performance for males: The dispersion parameter for negative binomial model, theta [SE], 1.89 [0.04]); Akaike information criterion 47,381. Model performance for females: The dispersion parameter for negative binomial model, theta [SE], 2.05 [0.03]); Akaike information criterion 69,274.

^#^The interaction analyses were conducted on the total sample to examine the potential moderating effects of sex on the relationship between each explanatory variable and the outcome. The statistically significant interactions indicated that sex plays an important role in influencing how the explanatory variables relate to the sick leave duration.

### Subgroup analyses for predicting sick leave duration (1–180 days)

Odds of longer sick leave duration increased if individuals were self-employed (OR 1.13), older (OR 1.00 per gained year), single with no children (1.07), on sick leave before COVID-19 (OR 1.12), required inpatient care (OR 1.31), and of the female sex (OR 1.02). Moreover, the odds of longer sick leave duration decreased if individuals had a medium income level (OR 0.97) and for those without laboratory confirmed COVID-19 virus (ICD code, U07.2 [OR 0.90]) (Supplementary Table 1). Results of the interaction analysis are presented in Supplementary Table 2.

## Discussion

In this Swedish nationwide registry study on sick leave during the first wave of the pandemic, people were on sick leave for a median of 5 weeks. Among the 11,902 individuals, 3% were still on sick leave after 12 months, with a slightly higher proportion among males. Moreover, sociodemographic characteristics, previous history of sick leave, and need for inpatient care due to COVID-19 were significantly associated with the duration of sick leave. These results reflected the multitude of factors that influenced sick leave and work capacity after COVID-19 and indicated the need for long-term follow-up^19^. In the present study, individuals with a longer duration of sick leave could have had a post COVID-19 condition, defined as symptoms that lasted for at least 12 weeks^20^. Therefore, person-centered rehabilitation might be necessary for people who have persistent symptoms and are unable to work.

The outcome of this study, sick leave durations from 1 to 365 days, revealed two peaks: one at 181 days and another towards the end of the study period. The first peak could be linked with the Swedish system regarding sick leave. According to Swedish sick leave regulations, till ≤ 180 days of sick leave, work ability was related to present employment. From ≥ 180 days, it was related to the general labor market. The second peak at 365 days corresponded to the termination of our study's follow-up period.

In the present study, individuals were more likely to have a longer sick leave duration if they were female, older, self-employed or unemployed, single (with or without children), or had a history of sick leave prior to COVID-19. However, individuals were less likely to have a longer sick leave duration if they did not require inpatient care, were born outside Sweden, or had a non-detected SARS-CoV-2 diagnosis (ICD code: U07.2). Generally, sick leave was more common among females than among males in the Swedish population, which was also reported in COVID-19 cohorts^7–9,21^. While females were more likely to report a greater degree of disability during the subacute and post-acute phases of COVID-19, males were more likely to have more severe COVID-19 and a greater requirement for inpatient care^2,13,14^. Individuals born outside of Sweden had a reduced likelihood of experiencing extended sick leave during the COVID-19 pandemic. While our regression model did not directly consider the birthplace's specific origin, our results parallel those found in the study by Spets et al.^22^. This implies that the country of birth, especially in the context of low-income countries, could exert a substantial influence on the duration of sick leave. Potential explanations for these findings could be the fear of losing employment or a pressing need to provide economic support to one's family^23^. Our findings could be interpreted regarding studies on sick leave due to other conditions and show the different aspects of importance in returning to work for females and males^24^.

In the presented study, older age was also found to predict longer sick leave duration in all analyses. However, the true effect of age was unclear. Older age was related to long-term sick leave owing to COVID-19^18^. However, previous findings suggested that a longer duration of sick leave was related to older age and severity of COVID-19 infection in females and males, respectively^16^. When interpreting the results of the present and previous studies, it must be considered that older males are more likely to develop severe COVID-19 and require inpatient care^14,25^.

In the total sample, as well as in the subgroup of males and females, not having detected SARS-CoV-2 virus was associated with lower odds for long sick leave duration. It could be speculated that individuals without detected virus had a less severe infection, as previous findings reported differences in self-reported symptoms after COVID-19 diagnosis based on the positive laboratory SARS-CoV-2 test^26^. However, these speculations should be handled cautiously as there was a shortage of test material in primary care during the first wave of the pandemic in Sweden, which led to U07.2 as a common diagnosis. It was possible that sick leaves were underestimated in this study. In this cohort, we previously showed that recurrent sick leave was present in 2.0% individuals within the first four months of sick leave (10). This may indicate that the number of people with longer sick leave due to COVID-19 may be higher than that in the present study.

Data from individuals who died during the first four months were not analyzed, which indicated that individuals with the most severe outcomes due to COVID-19 may not have been covered. The negative binomial regression models were fitted using a machine learning approach for two primary reasons. First, these models have the potential to generalize to new, unseen data, which is crucial in predicting outcomes across various populations. Second, machine learning models offer the advantage of being regularly updated as new data becomes available. This capability allows for the integration of the latest information and trends, a feature particularly vital in the rapidly changing landscape of a pandemic.

A limitation of this study was that we had no information on deaths beyond the first four months of follow-up. However, this large nationwide cohort constituted robust data with a presumed representative sample given its size and coverage. These results should be interpreted in relation to the Swedish context as the availability of paid sick leave may differ between countries^27–30^. Another limitation was that some variables that may be relevant when analyzing sick leave were lacking. We did not have information on the initial COVID-19 severity. However, information on the need for inpatient care was used as a proxy for COVID-19 severity. In addition, information on the type of work, work demands, and tasks was unavailable. However, we used employment types that provided work-related information to explain the sick leave duration. Nevertheless, this study lacked information on variables that may have contributed to explaining sick leave duration^31^.

## Conclusions

The results of this nationwide registry-based study indicated that three out of 100 individuals were still on sick leave one year after their COVID-19 diagnosis. Aspects of the importance of sick leave duration differed between males and females and comprised sociodemographic characteristics and the need for inpatient care. This indicated the complexity of sick leave due to COVID-19. These results indicate the need for long-term follow-ups to identify people who might benefit from targeted vocational rehabilitation.

### Supplementary Information

Supplementary Tables.

## Data Availability

Due to the sensitive nature of the data collected for this study, open access cannot be provided. Requests to access the dataset can be submitted from qualified researchers to the authors. According to the Swedish regulation http://www.epn.se/en/start/regulations/, data can only be used in accordance with the application for this study that was approved by the Ethical board.
