# Supplementary material for: Sick leave one year after COVID-19 infection: a nationwide cohort study during the first wave in Sweden
Source: Sci Rep. 2024 Jan 5;14:572. doi: 10.1038/s41598-023-50413-9 (PMC10766961; doi:10.1038/s41598-023-50413-9)
Supplement: Supplementary file 1 — Supplementary Tables. [file 41598_2023_50413_MOESM1_ESM.docx]

**Supplementary material**

Title: Sick leave during one year after COVID-19 infection, a nationwide cohort study during the first pandemic wave in Sweden.

Tamar Abzhandadze, OT, PhD^1,2*^, Emma Westerlind, MD, PhD^1,3^, Annie Palstam, PT, PhD^1,4^, Katharina S. Sunnerhagen, MD, PhD^1,4^, Hanna C. Persson, PT, PhD^1,2^.

^1^Institute of Neuroscience and Physiology, The Sahlgrenska Academy, University of Gothenburg, Gothenburg, Sweden.

^2^Department of Occupational Therapy and Physiotherapy, Sahlgrenska University Hospital, Gothenburg, Sweden.

^3^Department of Emergency medicine, Sahlgrenska University Hospital, Gothenburg, Sweden.

^4^ Department of Neurocare, Sahlgrenska University Hospital, Gothenburg, Sweden.

***Corresponding author:**Tamar Abzhandadze, PhD

Institute of Neuroscience and Physiology

Rehabilitation Medicine, University of Gothenburg

Per Dubbsgatan 14, fl. 3.

413 45 Gothenburg, Sweden

Telephone: +46 702 622 897

E-mail: [tamar.abzhandadze@gu.se](mailto:tamar.abzhandadze@gu.se)

ORCID: 0000-0002-0069-6875

Supplementary table 1. Multivariable negative binomial regression model explaining duration of sick leave (range 1-180 days) during first wave of COVID-19 infection.

| Variable names and categories | B | SE | OR | 95 % CI | | Adjusted  P-value |
| --- | --- | --- | --- | --- | --- | --- |
| Ref. Employed |  |  |  |  |  |  |
| Self-employment | 0.12 | 0.04 | 1.13 | 1.04 | 1.22 | <0.001 |
| Unemployment | 0.01 | 0.04 | 1.01 | 0.93 | 1.09 | 0.729 |
| Age (Range 18 -77) | 0.00 | 0.00 | 1.00 | 1.00 | 1.00 | <0.001 |
| Ref. Country of birth, Sweden |  |  |  |  |  |  |
| All other countries | -0.02 | 0.01 | 0.98 | 0.96 | 1.00 | 0.112 |
| Ref. Familial status, married no children |  |  |  |  |  |  |
| Married, with children | 0.02 | 0.02 | 1.02 | 0.98 | 1.05 | 0.308 |
| Single, with children | 0.03 | 0.02 | 1.03 | 0.99 | 1.07 | 0.195 |
| Single, no children | 0.07 | 0.02 | 1.07 | 1.00 | 1.11 | <0.001 |
| Ref. SARS-CoV-2 detected, U07.1 |  |  |  |  |  |  |
| SARS-CoV-2 not detected, U07.2 | -0.10 | 0.01 | 0.90 | 0.89 | 0.92 | <0.001 |
| Ref. Low income |  |  |  |  |  |  |
| Medium income | -0.03 | 0.01 | 0.97 | 0.94 | 1.00 | 0.002 |
| High income | 0.00 | 0.01 | 1.00 | 0.97 | 1.02 | 0.768 |
| Ref. No sick leave prior to COVID-19 |  |  |  |  |  |  |
| Sick leave prior to COVID-19, ≥28 days or ≥6 times | 0.11 | 0.01 | 1.12 | 1.09 | 1.14 | <0.001 |
| Ref. Education, ≤ 12 years |  |  |  |  |  |  |
| Education, ≥ 13 years | 0.02 | 0.01 | 1.02 | 1.00 | 1.04 | 0.098 |
| Ref. No inpatient care |  |  |  |  |  |  |
| Inpatient care | 0.27 | 0.01 | 1.31 | 1.28 | 1.34 | <0.001 |
| Ref. Male sex |  |  |  |  |  |  |
| Female | 0.02 | 0.01 | 1.02 | 1.00 | 1.04 | 0.044 |
| Statistics: negative binomial regression model developed on the test data set. The dispersion parameter for negative binomial model, theta [SE], 4.59 [0.07]); Akaike information criterion 77464. The RMSE 25.8, and variance of the model (R-squared) 0.04. Testing data set: RMSE 24.5; R-squared 0.06. Abbreviations: SARS-CoV-2, COVID-19 virus infection; Bootstrapped estimates: B, regression coefficient; SE, Standard Error; OR, Odds ratio; 95 % CI, 95 % confidence interval for odds ratio. ^#^The population size in regression analysis was 11 799 individuals due to missing data on explanatory variables. | | | | | | |

Supplementary table 2. Individual negative binomial regression analyses for male and female individuals. Interaction analyses on effects of Sex for explaining sick leave duration (range 1 – 180 days) during first wave of COVID-19.

| Explanatory variables | Males (n = 4344) ^#^  OR (95 % CI) | | | Females (n = 6702) ^#^ OR (95% CI) | | | 95 % CI*_Interaction_* | | P*_Interaction_* |  |
| --- | --- | --- | --- | --- | --- | --- | --- | --- | --- | --- |
| Ref. Employed |  |  |  |  |  |  |  |  |  |  |
| Self-employment | 1.05 | (0.95 | 1.16­) | 1.08 | (0.96 | 1.21) | 0.84 | 1.15 | 0.828 |  |
| Unemployment | 0.99 | (0.86 | 1.14) | 0.96 | (0.86 | 1.08) | 0.82 | 1.12 | 0.608 |  |
| Age (Range 18 -77 years) | 1.00 | (1.00 | 1.00)*** | 1.00 | (1.00 | 1.01)*** | 1.00 | 1.00 | 0.025 |  |
| Ref. Country of birth, Sweden |  |  |  |  |  |  |  |  |  |  |
| All other countries | 0.98 | (0.94 | 1.01) | 0.99 | (0.97 | 1.02) | 0.96 | 1.03 | 0.799 |  |
| Ref. Familial status, married no children |  |  |  |  |  |  |  |  |  |  |
| Married, with children | 1.02 | (0.96 | 1.08) | 1.02 | (0.98 | 1.07) | 0.97 | 1.09 | 0.269 |  |
| Single, with children | 1.02 | (0.94 | 1.10) | 1.03 | (0.98 | 1.08) | 0.92 | 1.13 | 0.697 |  |
| Single, no children | 1.06 | (1.00 | 1.13)* | 1.07 | (1.03 | 1.12)** | 0.95 | 1.07 | 0.663 |  |
| Ref. SARS-CoV-2 detected, U07.1 |  |  |  |  |  |  |  |  |  |  |
| SARS-CoV-2 not detected, U07.2 | 0.90 | (0.87 | 0.94)*** | 0.90 | (0.88 | 0.93)*** | 0.93 | 1.01 | 0.180 |  |
| Ref. Low income |  |  |  |  |  |  |  |  |  |  |
| Medium income | 0.95 | (0.91 | 0.99)** | 0.97 | (0.95 | 1.00)* | 1.00 | 1.08 | 0.032 |  |
| High income | 0.99 | (0.95 | 1.03) | 1.01 | (0.99 | 1.03) | 0.97 | 1.05 | 0.440 |  |
| Ref. No sick leave prior to COVID-19 |  |  |  |  |  |  |  |  |  |  |
| Sick leave before COVID-19, ≥28 d or ≥6 t | 1.12 | (1.05 | 1.18)*** | 1.13 | (1.09 | 1.17)*** | 0.92 | 1.04 | 0.619 |  |
| Ref. Education, ≤ 12 years |  |  |  |  |  |  |  |  |  |  |
| Education, ≥ 13 years | 0.98 | (0.94 | 1.02) | 1.04 | (1.01 | 1.07)* | 1.05 | 1.14 | <0.001 |  |
| Ref. No inpatient care |  |  |  |  |  |  |  |  |  |  |
| Inpatient care | 1.35 | (1.30 | 1.40)*** | 1.27 | (1.22 | 1.32)*** | 0.88 | 0.99 | 0.012 |  |
| Abbreviations: SARS-CoV-2, COVID-19 virus infection; OR, Odds ratio; 95 % CI, 95 % confidence interval for odds ratio. *** p <0.001, ** p< 0.01, * p <0.05. Statistics: negative binomial regression model. ^#^The number of individuals is different due to missing data on explanatory variables.  Model performance for males: The dispersion parameter for negative binomial model, theta [SE], 4.30 [0.10]); Akaike information criterion 38455. Model performance for females: The dispersion parameter for negative binomial model, theta [SE], 4.91 [0.09]); Akaike information criterion 58184 | | | | | | | | | | |
